# DNA-templated synthesis of biomimetic cell wall for nanoencapsulation and protection of mammalian cells

**DOI:** 10.1038/s41467-019-10231-y

**Published:** 2019-05-20

**Authors:** Peng Shi, Nan Zhao, James Coyne, Yong Wang

**Affiliations:** Department of Biomedical Engineering, The Pennsylvania State University, University Park, Pennsylvania, 16802 USA

**Keywords:** Biomaterials - cells, Bioinspired materials, Organizing materials with DNA

## Abstract

Mammalian cells are different from plant and microbial cells, having no exterior cell walls for protection. Environmental assaults can easily damage or destroy mammalian cells. Thus, the ability to develop a biomimetic cell wall (BCW) on their plasma membrane as a shield can advance various applications. Here we demonstrate the synthesis of BCW with a framing template and a crosslinked matrix for shielding live mammalian cells. The framing template is a supramolecular DNA structure. The crosslinked matrix is a polyelectrolyte complex made of alginate and polylysine. As the entire procedure of BCW synthesis is strictly operated under physiological conditions, BCW-covered mammalian cells can maintain high bioactivity. More importantly, the data show that BCW can shield live mammalian cells from not only physical assaults but also biological assaults. Thus, this study has successfully demonstrated the synthesis of BCW on live mammalian cells with great potential of shielding them from environmental assaults.

## Abstract (web summary)

The creation of protective barriers around cells is of interest for a range of applications. Here, the authors report on the creation of DNA templated alginate-polylysine biomimetic cell walls for encapsulating and shielding cells, demonstrating protection against physical assaults and immune reactions.

## Introduction

During evolution, most plant and microbial cells have developed an exterior cell wall that has a framing structure and a crosslinked matrix^1–5^. While this cell wall is an ultrathin nanomaterial, it can function as an effective shield to protect the cells from environmental assaults. Thus, plant and microbial cells can survive and recover even after their living environment is significantly changed from benign to harsh conditions^1–5^. Mammalian cells, however, do not have a cell wall. The outermost layer of a mammalian cell is only a delicate, two-layered structure of phospholipids with proteins embedded^6^. While the internal cytoskeleton mechanically helps mammalian cells maintain their shapes, mammalian cells can be easily damaged or destroyed when exposed to physical or biological assaults. Therefore, to advance the applications of mammalian cells, it is necessary to develop methods and materials to shield them externally.

Great effort has been made to deposit materials on the plasma membrane of mammalian cells for their protection^7–13^. The most common strategy is to drive the mixture of cells and polymers into another phase for gelation or solidification using fluidic devices^14,15^. For instance, the He group has applied this strategy to develop core–shell microcapsules for the differentiation and delivery of pluripotent stem cells for the treatment of myocardial infarction^16^. However, the cell flow to the nozzle of a device is an intrinsically random process determined by Poisson statistics, which leads to heterogeneous polymer deposition and poor quality control^17,18^. The deposited polymer usually has a very high volume, which can be several orders of magnitude higher than the cell. It causes poor molecular transport and cell survival^19–21^. Moreover, if an application requires in vivo cell transplantation, it is challenging for target tissues or organs to accommodate a high number of transplanted cells owing to the large volume of polymers^19–21^. To reduce the volume of depositing materials, methods have been developed to convert metal ions into a metallic nanomaterial with a redox reaction on the plasma membrane^22,23^. However, the reaction conditions are too harsh to maintain the viability of mammalian cells that have a very fragile cell membrane^9^. As the cell surface is negatively charged, studies have also been carried out to deposit cationic polymers or nanoparticles on the plasma membrane via electrostatic interactions^24–26^. However, positively charged polymers or nanoparticles are intrinsically cytotoxic^27–29^. Moreover, direct contact between the negatively charged cell membrane and positively charged molecules may turn on random endocytosis pathways for these materials to cross the cell membrane^29,30^. Thus, while the amount of depositing materials on the plasma membrane can be reduced, it remains challenging to apply these elegant methods to cover and shield live mammalian cells. Notably, all existing methods depend on the direct deposition of covering materials on mammalian cells. No method has been studied to generate a structure mimicking the exterior wall of plant and microbial cells.

DNA, polysaccharides and polypeptides are three essential polymers in nature. They and their synthetic counterparts have been widely used to build up materials for biological and biomedical applications^31–37^. For instance, two DNA hairpins can be used to synthesize long DNA polymers for biosensing through hybridization chain reaction (HCR)^38,39^. Alginate and polylysine, a polysaccharide and a polypeptide, can be crosslinked to form microcapsules for islet delivery through polyelectrolyte complexation (PC)^24,40,41^. We apply DNA, alginate and polylysine to synthesize a covering material to mimic the plant cell wall (i.e., BCW) on live mammalian cells.

**Fig. 1:**
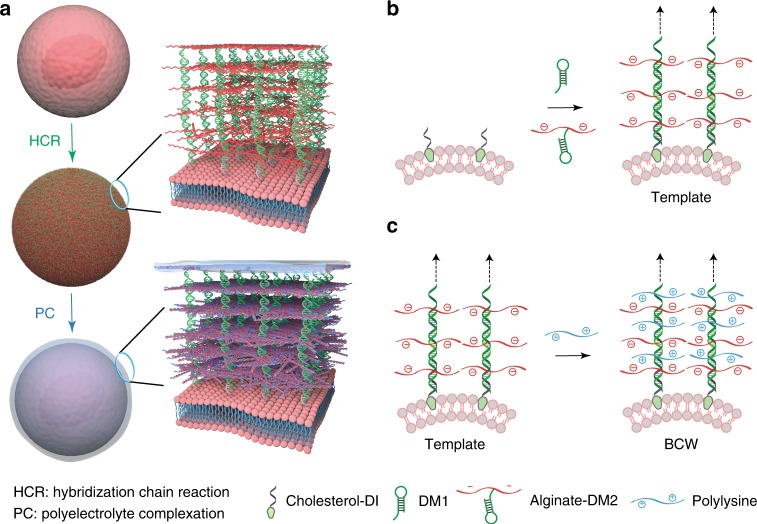
Schematic illustration of BCW synthesis. **a** Sequential HCR and PC on the live cell. HCR: hybridization chain reaction. PC: polyelectrolyte complexation. **b** Formation of the supramolecular DNA template and the concomitant DNA-templated alginate assembly during HCR. Cholesterol-conjugated DNA initiators (i.e., cholesterol-DIs) are inserted into the plasma membrane through cholesterol-lipid interactions. Cells with DNA initiators are incubated in the solution of DM1 and alginate-DM2 for the formation of the template on the plasma membrane. The arrows indicate multiple repeating units. **c** DNA-templated crosslinking of alginate and polylysine via PC

BCW has a framing template and a crosslinked matrix that are synthesized with HCR and PC, respectively (Fig. 1a). The framing template is a supramolecular DNA structure. It functions to direct molecular assembly and crosslinking of polysaccharides and polypeptides. Notably, as alginate is initially conjugated with one DNA hairpin to form an alginate-DNA macromer, the formation of the supramolecular template leads to automatic alginate assembly on the cell membrane (Fig. 1b) for the next-step polyelectrolyte complexation (Fig. 1c). Moreover, the entire procedure of BCW construction is conducted under physiological conditions. Thus, this method is promising for the synthesis of BCW in shielding live mammalian cells.

## Results

### DNA template-directed polymer assembly and crosslinking

**Fig. 2:**
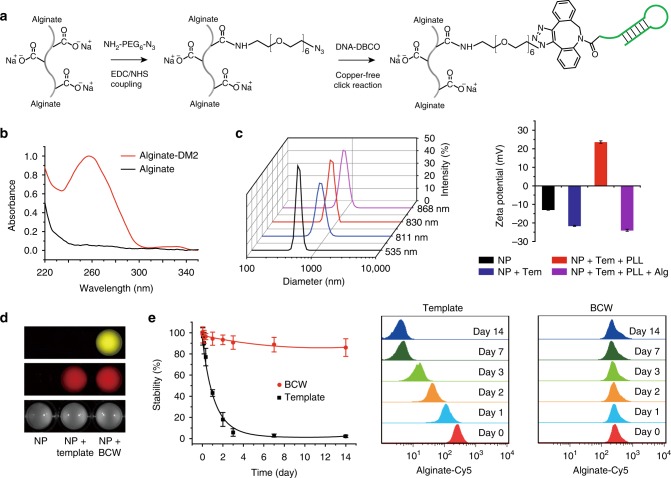
Formation of template and its directed polymer assembly and crosslinking. **a** Synthesis of alginate-DM2 macromer via click chemistry. **b** UV/Vis absorption spectra of alginate and alginate-DM2 macromer. **c** Size and zeta potential analysis of particles during the formation of the template and BCW using dynamic light scattering. Tem, template; PLL, polylysine; Alg, alginate. To explicitly show the generation of the template and BCW, 500 nm particles (NP) were used in the dynamic light scattering experiment during the construction of the template and BCW. All of other experiments were conducted with 5 μm particles to mimic cells unless otherwise noticed. Both particles were tethered with DNA initiators through the streptavidin-biotin interaction. **d** Fluorescence imaging of the particle suspension. Red: alginate-Cy5; yellow: polylysine-Cy3. **e** Examination of the stability of the template and BCW. Template-covered or BCW-covered particles were incubated in fetal bovine serum (10%) and then analyzed at predetermined time points with flow cytometry. Data are presented as mean ± standard deviation as indicated by error bars (*n* = 3)

We conjugated alginate and DNA to synthesize the alginate-DNA macromer using copper-free click chemistry (Fig. 2a). After conjugation, we characterized this macromer using UV spectroscopy and gel electrophoresis. The results demonstrate that these two polymers could be conjugated to form a hybrid macromer (Fig. 2b and Supplementary Fig. 1). However, as alginate and DM2 are both negatively charged, the presence of alginate in the macromer might repel intermolecular DM1–DM2 hybridization during HCR. Moreover, as alginate was conjugated with DM2, alginate might affect DM1–DM2 hybridization due to steric hindrance. Thus, it is important to examine whether two DNA hairpins can maintain the ability of hybridization for polymerization. The gel image (Supplementary Fig. 2) shows that DM1 and alginate-DM2 could polymerize, suggesting that steric hindrance and charge interactions did not significantly affect HCR. Notably, as alginate was conjugated to DM2, the result also suggests that the polymerization of the two DNA hairpins led to alginate assembly during the polymerization.

We further examined the polymerization of DM1 and alginate-DM2 on the DNA initiator-coated particles. Specifically, streptavidin-coated particles were sequentially treated with biotinylated DNA initiators and the solution of DM1 and alginate-DM2. The analysis of dynamic light scattering (DLS) showed that the radius of the particles was increased by ~140 nm (Fig. 2c). As the calculated length of one pair of DM1 and DM2 is approximately 16 nm, the increase of the particle size demonstrated that the polymerization of DM1 and alginate-DM2 led to the formation of the supramolecular DNA polymer, and more importantly that this supramolecular polymer functioned as a template to direct alginate assembly. Consistent with the DLS characterization, the zeta potential of the particles was decreased by ~70% owing to the increased density of negative charges on the particle surface (Fig. 2c).

As alginate assembled during the formation of the supramolecular DNA template, we anticipated that the template could further function as a framing structure to direct alginate-polylysine crosslinking. Indeed the treatment of the template-covered particles with polylysine led to an increase of the diameter and the reversal of the zeta potential (Fig. 2c). As alginate and polylysine were fluorescently labeled, we also imaged the particle suspension. The imaging analysis of the particle suspension is consistent with the measurements of size and zeta potential (Fig. 2d). These results further demonstrate that the supramolecular DNA template can form on the particle surface and also importantly this template can direct molecular assembly and crosslinking of alginate and polylysine through polyelectrolyte complexation to form BCW.

As BCW will be exposed to biological fluids in potential applications, we incubated template-coated or BCW-coated particles in serum to examine the stability of the template and BCW with flow cytometry. Based on the examination of the fluorescence signal of Cy5-conjugated alginate, the template-covered particles lost over 80% of fluorescence intensity within the first two days (Fig. 2e). By contrast, the BCW-covered ones maintained high fluorescence intensity during the two-week incubation after the initial decrease by 5–10% (Fig. 2e). The signal of FAM-labeled DM1 in the template also quickly decreased (Supplementary Fig. 3). However, while the signal of DM1 in BCW decreased, this decrease was much slower than that in the template. This observation is reasonable as the template is an uncrosslinked open system whereas BCW has the crosslinked alginate-polylysine complexes. The data also show that while the signal of DM1 in BCW was close to the background level in one week (Supplementary Fig. 3), the signal of alginate in BCW could be maintained over 90% (Fig. 2e). These data suggest that while the DNA template by itself or in BCW is degradable in biological fluids, the crosslinked alginate-polylysine cover once formed has high stability. The degradation of the DNA template after the construction of BCW may be beneficial to reduce potential inflammatory response caused by the interaction of DNA and toll-like receptors if BCW-covered cells will be used for in vivo cell transplantation.

### Synthesis of BCW on mammalian cells

**Fig. 3:**
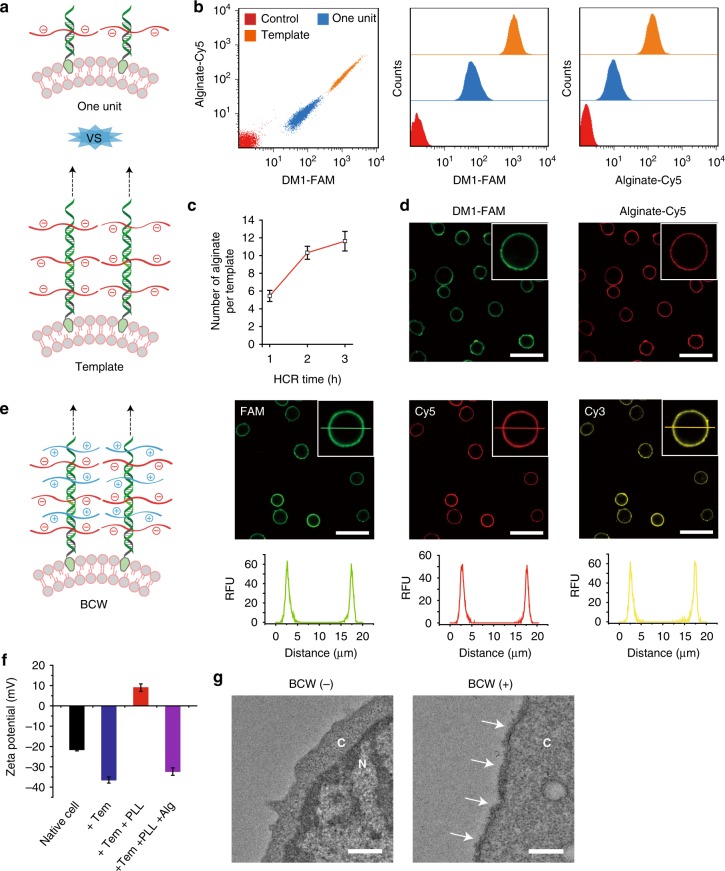
Synthesis and characterization of BCW on mammalian cells. **a** Schematic comparison between the framing template and the one-unit structure. **b** Examination of the template on the cell surface using flow cytometry. DM1 was labeled with FAM and alginate was labeled with Cy5. **c** Effect of reaction time on alginate assembly. **d** Confocal fluorescence images of the template on the cell surface. **e** Schematic illustration and confocal fluorescence images of BCW on live cells. DM1, alginate, and polylysine were labeled with FAM, Cy5, and Cy3, respectively. A representative line was drawn horizontally through the center of the cell for showing fluorescence distribution. Scale bar: 30 μm. **f** Zeta potential of the cell surface at each step during the formation of BCW. Tem, template; PLL, polylysine; Alg, alginate. **g** Examination of BCW on the cell surface using TEM. Cytosol is represented by C and nucleus is represented by N. BCW is indicated by the arrows. Scale bar: 500 nm. Data are presented as mean ± standard deviation as indicated by error bars (*n* = 3)

We next studied whether this method would be effective in synthesizing BCW on the plasma membrane of live mammalian cells using the CCRF-CEM cell line as the primary cell model. Cholesterol-conjugated DNA initiators were immobilized on the cell membrane through the insertion of cholesterol into the lipid bilayer. This insertion would be sufficient for the immobilization of DNA initiators and the initiation of HCR since membrane lipids are the major components in the plasma membrane. To examine the formation of the framing template on the cell surface (Fig. 3a), we used flow cytometry to analyze the signals of DM1 and alginate-DM2. Both of these signals exhibited a sharp shift (Fig. 3b). Importantly, the comparison between the one-unit and template groups demonstrates the success of DNA polymerization for the formation of the framing template on live cells (Fig. 3b). We further studied the effect of time on the formation of the framing template. The results suggest that within 3 h, one DNA template could grow to the level of displaying approximately 10 alginate molecules (Fig. 3c and Supplementary Fig. 4). The fluorescence imaging was consistent with the flow cytometry analysis, showing the strong signals of FAM and Cy5 localized on the cell membrane (Fig. 3d).

After demonstrating the formation of the template, we examined whether this template could direct the formation of BCW on live cells. The template-covered cells were incubated in the solution of Cy3-labeled polylysine for 1 min before centrifugation. The fluorescence images clearly show the strong Cy3 signals on the cell surface (Fig. 3e). Moreover, the same location on the cell membrane displayed the signals of three fluorescent labels for the DNA template, alginate and polylysine concomitantly (Fig. 3e). It is also important to note that the fluorescence intensity inside the cells was minimal compared to that on the cell membrane (Fig. 3e and Supplementary Fig. 5). These data clearly demonstrate that the template could direct alginate-polylysine crosslinking to form BCW on the cells, and that the cellular uptake of any molecule used for BCW synthesis was virtually negligible. We further quantified the yield of single cells after the formation of BCW. The percentage of single cells was over 90% (Supplementary Fig. 6). It suggests that the majority of BCW-covered cells were separate with minimal cell aggregation.

BCW was also examined using zeta potential and transmission electron microscopy (TEM). The negative zeta potential of the cells was decreased by 72% after the formation of the framing template (Fig. 3f). This negative zeta potential was reversed to 9 mV after template-directed alginate-polylysine complexation and a further treatment with alginate led to the reversal of the zeta potential (Fig. 3f). The TEM images suggest that the DNA-templated alginate-polylysine complexation led to the formation of BCW on the cell membrane with a thickness of approximately 70–150 nm (Fig. 3g and Supplementary Fig. 7). The ultrathin BCW ensures an ultralow polymer-to-cell ratio. It makes this method fundamentally different from other methods that need hydrogels of several hundred microns to cover cells^10^. Such a thick hydrogel cover is associated with problems such as poor molecular transport and high occupancy volume.

As the positively charged polylysine was directed to crosslink with the negatively charged alginate, one may suggest to directly cover the positively charged polylysine on the negatively charged cell surface. To address this potential concern and illustrate the advantage of our method, we incubated the cells in the solution of polylysine. The amount of polylysine on native cells was one order of magnitude lower than that of BCW (Supplementary Fig. 8a). It is also important to note that the distribution of polylysine on the cell membrane was heterogeneous and significantly scattered with large gaps (Supplementary Fig. 8b). Thus, while live cells can be directly treated with cationic polymers like polylysine^24,42^, it may be challenging to solve this problem of highly heterogeneous polymer distribution, let alone high cytotoxicity^27,28^. In contrary, our method spares the negatively charged cell surface from this direct deposition of cationic polylysine. With this method, we can generate a negatively charged supramolecular template that is able to direct the complexation between polylysine and alginate.

To further examine the effectiveness of our method in producing BCW, we quantified the amount of DNA initiators on the cell surface. This amount increased with the incubation time and the concentration (Supplementary Fig. 9). When the cells were incubated in the solution with 1 μM of DNA initiator, the surface of one cell displayed approximately 4 × 10^6^ DNA initiators. Supposed that each DNA initiator formed one template, the average distance between two DNA templates was 8.2 nm (Supplementary Table 1). Considering that the DNA template was approximately 140 nm in height (Supplementary Fig. 10) and 68 nm in hydrodynamic diameter (Supplementary Fig. 11), the whole cell surface would be adequately covered by supramolecular DNA templates for the alginate-polylysine complexation in the synthesis of BCW.

We also characterized molecular transport across BCW and the stability of BCW on the cell surface. Molecular transport was examined by incubating cells in the solution of FITC-labeled anti-CD4 antibody that can recognize and bind CCRF-CEM cells. The data show that while BCW was permeable to the antibody, it significantly blocked antibody transport in comparison to the template or the naked cell membrane (Supplementary Fig. 12). The stability was examined in two situations. One group of BCW-covered cells were cultured in a normal culture medium (10% FBS), and the other group of BCW-covered cells were cultured in a reduced-serum medium (1% FBS). With the treatment of 10% FBS, the fluorescence intensity of BCW on the cell surface decreased after 24 and 48 h (Supplementary Fig. 13). With the treatment of 1% FBS, the fluorescence intensity of BCW on the cell surface barely changed during the 48 h culture (Supplementary Fig. 13). As CCRF-CEM cells are divided faster in 10% FBS than in 1% FBS, these data suggest that BCW had high stability against enzymatic degradation, and that BCW-covered cells could maintain their normal capability of cell proliferation. Moreover, as the signal of alginate-Cy5 primarily came from the surface rather than the inside of the cells, the data suggest that BCW was not internalized during the procedures of BCW construction and cell culture.

**Fig. 4:**
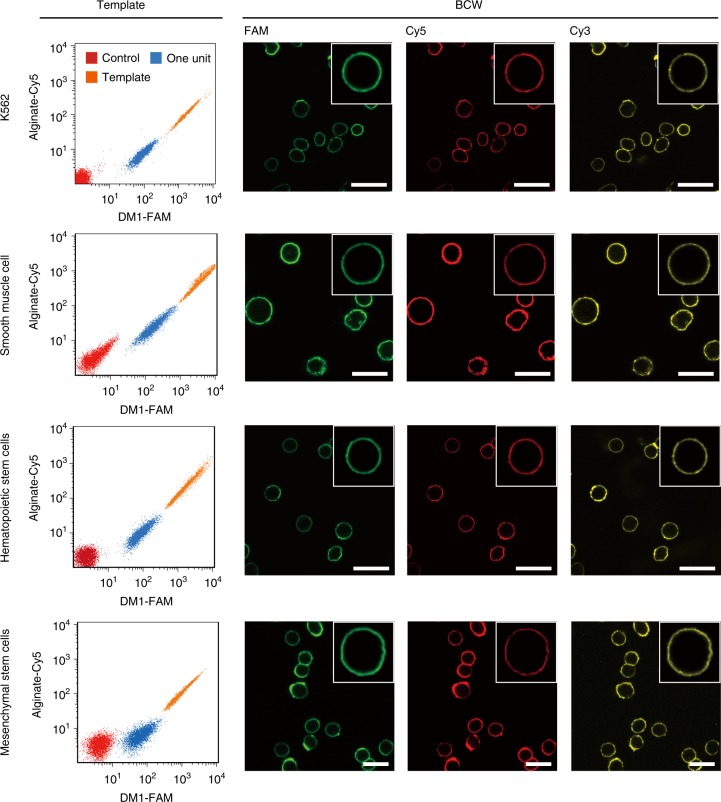
Formation of BCW on different types of cells. **a** Flow cytometry analysis of the template. **b** Confocal fluorescence images of BCW. Scale bar: 30 μm. FAM: DM1-FAM; Cy5: alginate-Cy5; Cy3: polylysine-Cy3

After the synthesis and characterization of BCW on CCRF-CEM cells, we further synthesized BCW on the membrane of additional four types of live mammalian cells using the exactly same protocol. BCW successfully formed on the cell membrane in all cases (Fig. 4 and Supplementary Fig. 14), although we did not optimize reaction conditions for each different cell type. Together, the data demonstrate that DNA-templated molecular assembly and crosslinking is an effective, potentially universal method for the synthesis of BCW on the plasma membrane of live mammalian cells.

### Evaluation of shielding effectiveness

**Fig. 5:**
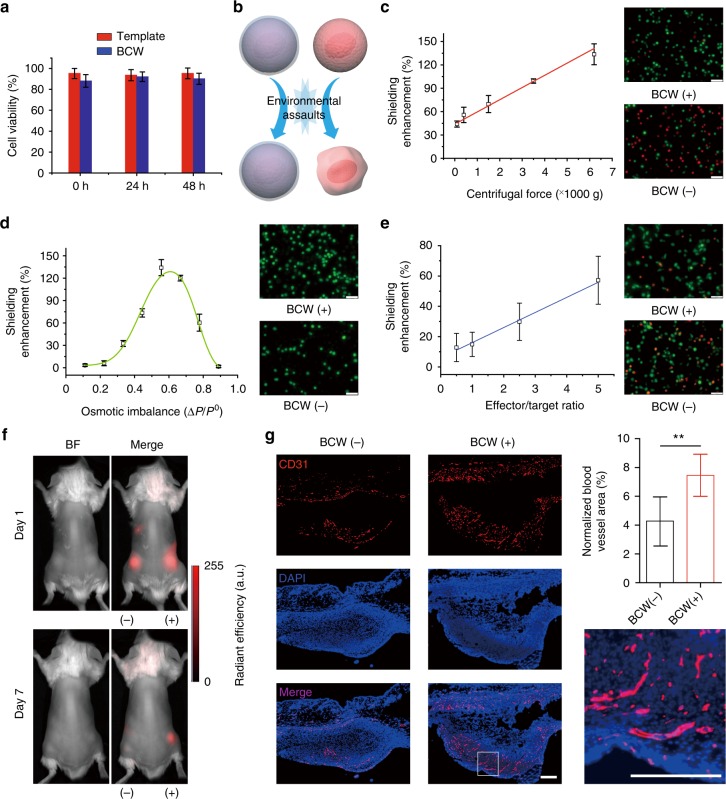
Evaluation of shielding enhancement. **a** Examination of cell viability at different time points. The viability of native cells was used as 100%. 0 h: analysis of the cells right after the synthesis of the template or BCW. **b** Schematic comparison of the cells covered with (bottom) or without (top) BCW when exposed to environmental assaults. **c** Shielding enhancement-centrifugal force relationship. After the assault, the cells were cultured and examined with the LIVE/DEAD staining. Green: live; red: dead. Representative images were taken after the cells were exposed to the centrifugal force of 6200×*g*. **d** Shielding enhancement-osmotic imbalance relationship. The cells were stained with Calcein-AM (green) for imaging before exposed to the conditions of osmotic imbalance. Representative images were taken after the cells were incubated in the solution of 0.4% NaCl. CCRF-CEM was used for Fig. 5a–d. **e** Shielding enhancement-immune attack relationship. K562 and NK-92MI were used as target and effector cells, respectively. Representative images were taken at the 2.5:1 effector/target ratio. Green: CFSE. Red: dead cells. Scale bars: 50 μm. Data are presented as mean ± standard deviation as indicated by error bars (*n* = 3). **f** In vivo imaging of human bone marrow MSCs subcutaneously transplanted into the mice. MSCs expressed red fluorescence protein (RFP). BF, bright field. (−): naked cells (without BCW); (+): BCW-covered cells. Color-coding of fluorescence intensity in arbitrary units (a.u.) is equally scaled for the images. **g** Staining of endothelial cells using a mouse-specific anti-CD31 antibody. **p < 0.01. The paired Student’s *t*-test was used to compare the two groups. Scale bars: 200 μm. Data are presented as mean ± standard deviation as indicated by error bars (*n* = 6)

After demonstrating the formation of BCW on mammalian cells, we studied the effect of BCW on cell viability. The inability to maintain high cell viability is a major reason for the failure of many traditional methods for cell surface engineering and/or shielding. For instance, the direct deposition of cationic polymers on the naked cell membrane is well-known to cause high cytotoxicity^27,28^. In our method, a framing template was synthesized on the cell membrane before the treatment of cells with polylysine. Polylysine would mainly react with DNA-templated alginate, sparing the plasma membrane of cells. Moreover, the entire procedure of BCW formation did not involve any harsh conditions. Indeed BCW-covered cells could maintain much higher viability than those naked cells directly exposed to polylysine (Fig. 5a and Supplementary Fig. 15).

As BCW was developed for cell protection, we examined the effectiveness of BCW in shielding mammalian cells from physical and biological assaults (Fig. 5b). Reiterative centrifugation and osmotic imbalance were used as physical assaults. The results show that naked cells were highly sensitive to centrifugation (Supplementary Fig. 16). For instance, when the centrifugal force was as low as 110 g, over 30% of the cells lost cell viability during the procedure of cyclic washing and centrifugation. By contrast, even though the centrifugal force was increased over 6000 × *g*, approximately 85% of the cells could maintain viability (Supplementary Fig. 16). Thus, these data clearly demonstrate that BCW can shield live cells from the damage of centrifugation (Fig. 5c). The shielding enhancement increased linearly with the centrifugal force (Fig. 5c), suggesting that live cells can be protected more effectively in a harsher centrifugation situation. We also examined the effect of osmotic imbalance on the cell viability. The shielding enhancement-osmotic imbalance relationship exhibits a bell curve (Fig. 5d). This relationship suggests that when the osmotic imbalance changed from 0.1 to 0.6, BCW was strong enough to protect the covered cells. With a further increase of the osmotic imbalance, BCW could still play the role of shielding the cells while this shielding effect started to weaken (Fig. 5d and Supplementary Table 2).

In addition to the two physical assaults, we also studied cell viability after the cells were exposed to biological assaults using both in vitro and in vivo assays. In the in vitro assay, a natural killer cell line (NK-92MI) was used to attack BCW-covered K562 cells. The results show that BCW enhanced the survival rate of the target cells from the assault of NK-92MI virtually linearly (Fig. 5e and Supplementary Fig. 17). For instance, when the effector/target ratio was 5:1, ~50% of target cells lost viability whereas ~85% of BCW-covered cells could maintain viability (Supplementary Fig. 17). In the in vivo assay, we subcutaneously transplanted human bone marrow mesenchymal stem cells (MSCs) expressing red fluorescence protein (RFP) into BALB/c mice. As foreign cells, human MSCs would be quickly destroyed in mice through the host immune response. As indicated by the signal of RFP, we could initially detect MSCs covered with (+) or without (−) BCW. However, the RFP signal could be barely detected in the mice transplanted with naked MSCs (i.e., no BCW) at day 7. In contrast, the RFP intensity maintained 30% in the mice transplanted with BCW-covered MSCs (Fig. 5f and Supplementary Fig. 18). So the sharp difference strongly demonstrates that BCW can shield human MSCs from biological assaults in the in vivo environment. To better understand this shielding effect, we also conducted two in vitro experiments. In the first one, we incubated BCW-covered MSCs on the cell culture plate. The results demonstrate that adherent MSCs could maintain viability, break BCW and attach to the cell culture plate for proliferation (Supplementary Fig. 19). In the second one, we transferred the fibrin hydrogel with MSCs onto the cell culture plate. The results show that MSCs covered with or without BCW did not migrate from the fibrin hydrogel to the outside (Supplementary Fig. 20), suggesting that the disappearance of MSCs from the implantation site in mice did not result from MSC migration. As it is known that MSCs can induce the growth of blood vessels because of their ability of releasing angiogenic factors^43,44^, in another in vivo assay, we transplanted MSCs into mice for examining angiogenesis. The tissues at the transplantation sites were collected and stained with a mouse-specific anti-CD31 antibody as CD31 is a typical endothelial cell biomarker. The data show that BCW-covered MSCs could stimulate more angiogenesis than the naked cells (Fig. 5g and Supplementary Fig. 21). Taken together, both in vitro and in vivo data demonstrate that BCW holds great potential to cover and shield mammalian cells from environmental assaults.

## Discussion

A cell wall is not a simple ultrathin polymer layer but a structured polymer matrix crosslinked by microfibrils^1–5^. Because of this organized structure, while the cell wall is thin, it is mechanically tough with the ability to shield the cell. For instance, a major function of the cell wall is to prevent the over-swelling of the cell when water enters under osmotic imbalance^1,5^. We mimicked the key features of the natural cell wall to develop BCW on mammalian cells. BCW has a supramolecular DNA frame that guides the formation of the polysaccharide-polypeptide matrix. Notably, while we used alginate and polylysine to illustrate the formation of the matrix, in principle, they can be replaced with any other biocompatible polymers that can undergo crosslinking reactions including but not limited to polyelectrolyte complexation.

The synthesis of BCW is fundamentally different from previous methods studied for the deposition of materials on a surface. Firstly, with previous methods, covering materials are directly deposited on the plasma membrane without a framing structure and a structured matrix. Secondly, the entire procedure of BCW synthesis is strictly operated under physiological conditions. It does not involve any harsh conditions like organic solvents, high pressure, high salts, chemical crosslinking, or direct cell-toxic polymer contact, which are often used in other methods. For this reason, the BCW-covered cells can maintain high bioactivity after BCW synthesis, which is critical to any live mammalian cell-based applications. Thirdly, the volume of BCW is several orders of magnitude lower than that of the covered cell. The ultralow polymer-to-cell ratio would be beneficial if an application (e.g., in vivo transplantation) requires a high number of cells but a small space of occupancy. Lastly, the synthesis of BCW is simple and straightforward as it only needs cell incubation in biocompatible solutions. The formation of the template and the crosslinking of alginate and polylysine can both automatically happen in one single step. It does not need any specific devices or take much time or reiterative operations to accomplish. It is independent of cell type. Moreover, all cells are treated equally without an inherently theoretical difference. No empty BCW is generated. As a result, this method is suitable for the large-scale synthesis of BCW on a large number of cells. Therefore, we envision that BCW holds great potential as a shielding material for various live mammalian cell-based applications.

Cell shielding or encapsulation is needed in various areas^45^. Numerous elegant methods for cell shielding or encapsulation have been studied with great promise. Potential applications include cell delivery for tissue regeneration, cancer therapy, biosensing, bioprinting, etc^7,10,12,19,45,46^. However, while all of these applications require cell shielding or encapsulation, they do not share the same requirements for the shielding methods or materials. For instance, when cells are delivered into a target tissue for tissue repair via the release of therapeutic growth factors, it is unnecessary and undesirable to shield those therapeutic cells permanently. Similarly, when cells are mechanically extruded for three-dimensional bioprinting, temporary cell shielding would be sufficient to protect them against shear stress or microenvironmental damage during the procedure of extrusion.

Our work shows that BCW can protect cells against various environmental assaults. Moreover, BCW-covered cells can survive and break BCW for proliferation during cell culture. It suggests that BCW would be a promising tool for applications such as cell delivery for tissue regeneration and bioprinting. It is also important to note that other applications may require “permanent” shielding or encapsulation. A typical example is islet delivery for diabetes treatment. Such an application requires the shielding materials to have long-term stability and integrity (i.e., no breakage) and meanwhile allow sufficient molecular transport to support cell survival. While this work is not focused on the optimization of BCW for those applications, it may be possible to tune the conditions of BCW synthesis to achieve high stability and to provide cells or islets with a “permanent” shielding cover. It will be one of the foci in our future work.

## Methods

### Materials and instrumentation

Dibenzocyclooctyne-PEG_4_-NHS ester, Cy5-DBCO and Cy3 NHS Ester were purchased from Click Chemistry Tools (Scottsdale, AZ). Oligonucleotides (Supplementary Table 3) were purchased from Integrated DNA Technologies (Coralville, IA). Sodium alginate (medium viscosity, 80–120 kDa) and *O*-(2-Aminoethyl)-*O*′-(2-azidoethyl)pentaethylene glycol (NH_2_-PEG_6_-N_3_) were purchased from Sigma-Aldrich (St. Louis, MO). Streptavidin coated particles were purchased from Spherotech (Lake Forest, IL). PEGylated polylysine (26 kDa) was purchased from Nanosoft Polymers (Lewisville, NC). Quantum™ FITC-5 MESF was purchased from Bangs Laboratories (Fishers, IN). Carboxyfluorescein succinimidyl ester (CFSE) and Live/Dead viability/cytotoxicity kit were purchased from Invitrogen (Carlsbad, CA).

^1^H nuclear mgnetic resonance (NMR) was performed on a Bruker 500 MHz NMR spectrometer. UV–Vis absorption spectra were recorded using a Thermo Scientific NanoDrop 2000c spectrophotometer. The gel electrophoresis was run at 80 V for 20 min and the images of gels were recorded using a CRI Maestro EX System (Woburn, MA). Flow cytometry analysis was performed using a guava easyCyteTM flow cytometer (Millipore). FEI Talos F200X High-resolution Transmission Electron Microscope was used to record TEM images. Malvern Zetasizer Nano ZS was used to do size and zeta potential analysis. Olympus IX73 inverted microscope was used to record cell images. Olympus Fluoview 1000 was used to record confocal fluorescence images.

### General cell culture conditions

CCRF-CEM (CCL-119, human T lymphoblastic leukemia cell line) and K-562 (CCL-243, chronic myelogenous leukemia cell line) were purchased from ATCC (Manassas, VA) and maintained in RPMI-1640 supplemented with 10% FBS. NK-92MI (natural killer cell) was purchased from ATCC (Manassas, VA) and maintained in alpha minimum essential medium with recommended supplements. Primary Aortic Smooth Muscle Cells were purchased from ATCC (Manassas, VA) and maintained in M231 containing Smooth Muscle Cell Growth Supplement. Human bone marrow CD34+ hematopoietic stem cells were purchased from StemCell Technologies (Tukwila, WA,) and expanded with StemSpan serum-free expansion medium supplemented with CC100 cytokine supplements. hMSC (normal human bone marrow derived mesenchymal stem cells) was purchased from Lonza (Walkersville, MD) and maintained in recommended growth medium (Lonza). RFP-Tagged human bone marrow derived MSCs (RFP-MSCs) were purchased from Angio-Proteomie (Boston, MA) and maintained in stem cell growth medium (Lonza). Cells were maintained at 37 °C in an atmosphere of 5% CO_2_ and 95% relative humidity.

### Preparation of dibenzocyclooctyne-modified DNA (DNA-DBCO)

Hundred microliter of DNA-NH_2_ solution (1 mM) was added to 375 µL of modification buffer (DPBS, 50 mM NaHCO_3_). Then 25 µL of DBCO-PEG_4_-NHS ester (DMSO, 30 mM) was added and allowed to react for 6 h. It was repeated twice. The product DNA-DBCO was collected and purified using a 3 kDa Amicon Ultra Centrifugal Filter.

### Preparation of azide-modified alginate (alginate-N_3_)

Fifty milligram sodium alginate was dissolved in 5 mL of MES buffer (50 mM, pH = 5). To this solution, NHS (14 mg, 0.12 mmol), EDC (116 mg, 0.60 mmol) and NH_2_-PEG_6_-N_3_ (28 mg, 0.08 mmol) were added. After stirring for 30 min at room temperature, 55 µL of 6 M NaOH was added to adjust pH to 7.5–8.0. The reaction proceeded overnight at room temperature. Purification was achieved by 3 days of dialysis against water (10,000 MWCO membrane). To further remove any unreacted reagents, the alginate-N_3_ solution was precipitated in cold acetone, filtered and dried. The final product alginate-N_3_ was dissolved in d.i. H_2_O, filtered through a 0.22 μm membrane, and lyophilized. Alginate-N_3_ was analyzed using NMR spectroscopy.

### Preparation of alginate-DM2 macromer

The alginate-DM2 was prepared using copper-free click reaction by mixing alginate-N_3_ and DNA-DBCO. In brief, 100 µL of 1% w/v solution of alginate-N_3_ was incubated with 30 µL of 1 mM DNA-DBCO for 4 h at 37 °C. After conjugation, the alginate-DM2 was collected and purified using a 100 kDa Amicon Ultra Centrifugal Filter. For Cy5 modification, 100 µL of 1% w/v solution of alginate-DM2 was mixed with 30 µL of 1 mM Cy5-DBCO for 2 h at 37 °C. Gel electrophoresis and UV–Vis spectroscopy were used to confirm the successful conjugation.

### Quantitative analysis of alginate-DM2 macromer

To a solution containing 1% w/v alginate, variable concentrations of DM2 were added (50, 100, 150, 200, and 400 μM) and the absorption spectra of the different solutions were recorded. The increase in the absorbance at *λ* = 260 nm corresponded to the amount of DM2. A calibration curve relating the absorbance features of the systems as a function of DM2 concentration was generated. Then, 1% w/v of alginate-DM2 was prepared and the absorbance at *λ* = 260 nm was recorded. Based on the calibration curve, the DM2 concentration in 1% w/v alginate-DM2 solution was evaluated spectroscopically. Based on this quantitative analysis, 2–3 DNA molecules were conjugated to one alginate polymer chain.

### Synthesis of BCW on the particle surface

Two particles with 500 nm and 5 μm in diameter, respectively, were used for the examination of BCW on the particle surface. To clearly show the generation of the nanoscale template and BCW, we used 500 nm particles for the analyses of size and zeta potential. All of other experiments were conducted with 5 μm particles unless otherwise noticed. Streptavidin-coated particles (500 nm) (1 mg) were mixed with biotinylated DNA initiator (DI, 2 nmol) in 500 µL of PBS buffer at room temperature for 1 h on a rotator. DI-modified particles were collected by centrifugation and further washed with PBS. To examine the polymerization of DM1 and alginate-DM2 on the particles, 0.1 mg of particles were incubated in 800 µL of PBS containing DM1 (1 µM) and alginate-DM2 (1 µM) for 3 h at room temperature. To examine polyelectrolyte complexation, the particles with the template were incubated with 0.01% (w/v) polylysine in PBS for 5 min. After centrifugation and washing with PBS twice, the particles were further incubated with 0.05% (w/v) alginate in PBS for 5 min. For each step, the size and zeta potential of particles were monitored using Malvern Zetasizer Nano ZS.

### Examination of BCW stability

DM1 was labeled with FAM. Alginate-DM2 was labeled with Cy5. The analysis of BCW stability was performed by tracking the changes of FAM and Cy5 signals as the function of time. The procedure of synthesizing the template and BCW was the same as described above for the analysis of size change and zeta potential except that 5 μm particles were used to mimic cells. Particles were incubated in the 10% fetal bovine serum (FBS)-supplemented RPMI 1640. An aliquot of particle solutions was removed at predetermined time points. After washed with DPBS, they were analyzed using flow cytometry. Experiments were performed in triplicate.

### Synthesis of BCW on live mammalian cells

CCRF-CEM cells were centrifuged, washed twice and re-suspended in DPBS. 1 × 10^6^ of cells were incubated in 400 µL of Cholesterol-TEG-DI solution (1 μM, DPBS) for 30 min for the incorporation of DI into cell membrane through the spontaneous insertion of cholesterol into membrane lipids. Then DI-modified cells were collected, washed and subsequently mixed with DM1 (1 μM) and alginate-DM2 (1 μM) in DPBS for 3 h to form the supramolecular DNA template. To test the effect of reaction time on the formation of the template on the cells, the polymerization time was varied from 1 to 3 h. The cells were collected and the fluorescence intensity (Cy5 signal from alginate-DM2) was measured by flow cytometry. For the subsequent polyelectrolyte complexation, cells covered with the template were sequentially treated with 0.01% (w/v) polylysine for 1 min and 0.05% (w/v) alginate for 5 min. Finally, the cells covered with BCW were collected by centrifugation before any characterization.

### Transmission electron microscopy (TEM)

For the preparation of TEM samples, naked or BCW-covered cells were fixed in the solution of glutaraldehyde (2.5%). The cell samples were then post-fixed with 1% osmium tetroxide. After washed with cacodylate buffer (0.1 M), the cells were dehydrated with a series of ethanol solutions (30, 50, 70, 90, and 100%). All of these treatments were performed at 4 °C. After that, the cells were treated with propylene oxide, infiltrated and embedded in a liquid resin. The resin block was sectioned using an ultramicrotome and the slices were collected on grids. Imaging was performed under the FEI Talos F200X High-resolution transmission electron microscope using both TEM and STEM modes.

### Quantitation of DI on the cell surface

To test the effect of incubation time on the display of DI on the cell surface, 5 × 10^5^ of cells were incubated in 400 µL of Cholesterol-TEG-DI solution (1 µM, DPBS) at room temperature for a different period of time. To test the effect of DI concentration on the display of DI on the cell surface, 5 × 10^5^ of cells were incubated with Cholesterol-TEG-DI (0.1–10 µM) in DPBS for 30 min. DI-modified cells were collected, washed and subsequently stained with DM1-FAM (1 µM). The mean fluorescence intensity (MFI) of cells in each group was measured by flow cytometry and compared with the known standards (Quantum™ FITC MESF, Bangs Laboratories) to determine the number of DI per cell.

### Examination of antibody transport across BCW and stability of BCW

To test molecular transport, cells were incubated with anti-human CD4-FITC (Thermo Fisher, # 11-0049-42) in DPBS (1:200 dilution) at 37 °C for 10 min. Then cells were washed with DPBS for three times. The binding of anti-CD4 was analyzed using both flow cytometry and fluorescence imaging. The nuclei of cells were stained with DAPI.

To test the stability, BCW-covered cells were treated with FBS in two situations. One group of cells were cultured in a normal culture medium (10% FBS-supplemented RPMI 1640), and the other group of cells were cultured in a reduced-serum medium (1% FBS-supplemented RPMI 1640). The purpose of using the normal cell culture medium was to evaluate whether the covered cells could undergo cell survival, attachment and proliferation. The purpose of using the reduced-serum medium was to evaluate if biological fluids by themselves could degrade BCW. At predetermined time points, cells were analyzed using flow cytometry and fluorescence imaging.

### Evaluation of shielding enhancement

Centrifugal force and osmotic imbalance were used to demonstrate the effects of physical assaults on cells. In the study with the centrifugal force, cells covered with or without BCW were suspended in the DPBS at a concentration of 1 × 10^6^ cells/mL. The centrifugation was repeated by five times. Each centrifugation was performed for 5 min at 4 °C. The centrifugation force was varied from 110 to 6200 g. Cells were re-suspended in new DPBS after each centrifugation. After five times of centrifugation, the cells were incubated in the culture media for 24 h and their viability was measured by Live/Dead viability/cytotoxicity kit. In brief, cells were incubated with calcein-AM and ethidium homodimer-1 at a concentration of 1 µM each. After 15 min incubation at room temperature in the dark, cells were imaged using fluorescence microscopy. Live (green) and dead (red) cells were counted using ImageJ. The viability was reported as the ratio of living cells to total cells.

Mammalian cells swell under hypotonic conditions to rupture their plasma membrane. To evaluate the response of cells to osmotic imbalance, cells (1 × 10^6^ cells/mL) covered with or without BCW were stained with calcein-AM and re-suspended in a series of NaCl solutions (0.1–0.8%). After 10 min incubation, cells were imaged using fluorescence microscopy. The cells with ruptured membrane were barely imaged under the microscope. So the cells maintaining integrity (green fluorescence) were counted. The viability was reported as the ratio of the cells with integrity to total cells. The BCW-mediated shielding enhancement was determined using Eq. 1:

1 $${\mathrm{\% }}\;{\mathrm{Shielding}}\;{\mathrm{enhancement}} = \left( {N_{{\mathrm{BCW}}} - N_{{\mathrm{native}}}} \right)/N_{{\mathrm{native}}}\;\times\;100$$

where *N*_BCW_ denotes the number of viable BCW-covered cells and *N*_native_ denotes the number of viable native cells.

Biological assaults were studied both in vitro and in vivo. The co-culture of K562 cells and immune effector cells is a common assay to examine immune attack. Thus, in the in vitro assay, target cells (K562 cells) were labeled by incubation with 1 μM CFSE for 5 min. The labeled cells were further covered with BCW according to the standard procedure described above. Target cells were transferred into a 48-well plate at 1 × 10^5^ cells/well. Immune effector cells (NK-92MI cells) were added to each well at varied effector/target ratios. The final volume was adjusted to 400 μL. The co-culture of K562 cells with NK-92MI cells was maintained at 37 °C for 3 h. After that, propidium iodide (PI) was added to each sample and incubated in the dark for 15 min to label dead cells. Cells were imaged using fluorescence microscopy. CFSE labeled (green) and PI labeled (red) cells were counted using ImageJ. The cell viability was determined by (Nc-Np)/Nc, where Nc and Np indicate the number of CFSE-positive cells and the number of PI-positive cells, respectively.

In the in vivo assay, RFP-hMSCs cells were subcutaneously transplated into BALB/c mice (age of 6–8 weeks). The animal study was performed according to the protocol approved by the Institutional Animal Care and Use Committee (IACUC). The use of hMSCs was approved by the Institutional Biosafety Committee (IBC). To avoid cell dissemination after transplantation, cells were transplanted with fibrinogen. In brief, 1 × 10^6^ cells were mixed with 50 µL of fibrinogen solution (20 mg/mL) and 50 µL of thrombin solution (2 U/mL) and injected subcutaneously to the dorsal flank of mice. The mice were imaged at day 1 and 7 after cell transplantation using the Maestro In Vivo Imaging System (CRI, Woburn, MA). Color-coding of fluorescence intensity in arbitrary units (a.u.) is equally scaled for all images. As MSCs can release signaling molecules to induce the growth of blood vessels, we also compared MSCs with or without BCW in inducing the growth of blood vessels. 1 × 10^6^ human MSCs were injected to the dorsal flank of mice. Mice were sacrificed with CO_2_ asphyxiation at day 10. The tissue in the transplantation site was harvested, fixed in 4% paraformaldehyde solution, blocked into paraffin, and sectioned (5 µm thickness). The sectioned tissues were deparaffinized, boiled in sodium citrate buffer, blocked with 3% BSA solution, and treated with rabbit anti-mouse CD31 antibody (Cell Signaling, #77699, 1:200 dilution) followed by incubation with Cy5-Goat anti-Rabbit secondary antibody (Thermo Fisher, # A10523, 1:200 dilution). The tissues were mounted in SlowFade Diamon Antifade Mountant with DAPI. The fluorescent images were taken under an Olympus IX73 microscope (Center Valley, PA). The CD31 positive areas were analyzed using ImageJ. The paired Student’s *t*-test was used to compare the two groups.

## Supplementary information

supplementary information

## Data Availability

All data are available from the authors on reasonable request.
