# Supplementary material for: DNA-templated synthesis of biomimetic cell wall for nanoencapsulation and protection of mammalian cells
Source: Nat Commun. 2019 May 20;10:2223. doi: 10.1038/s41467-019-10231-y (PMC6527693; doi:10.1038/s41467-019-10231-y)
Supplement: Supplementary file 1 — supplementary information [file 41467_2019_10231_MOESM1_ESM.docx]

**Supplementary Information**

**DNA-templated Synthesis of Biomimetic Cell Wall for Nanoencapsulation and Protection of Mammalian Cells**

Shi et al.


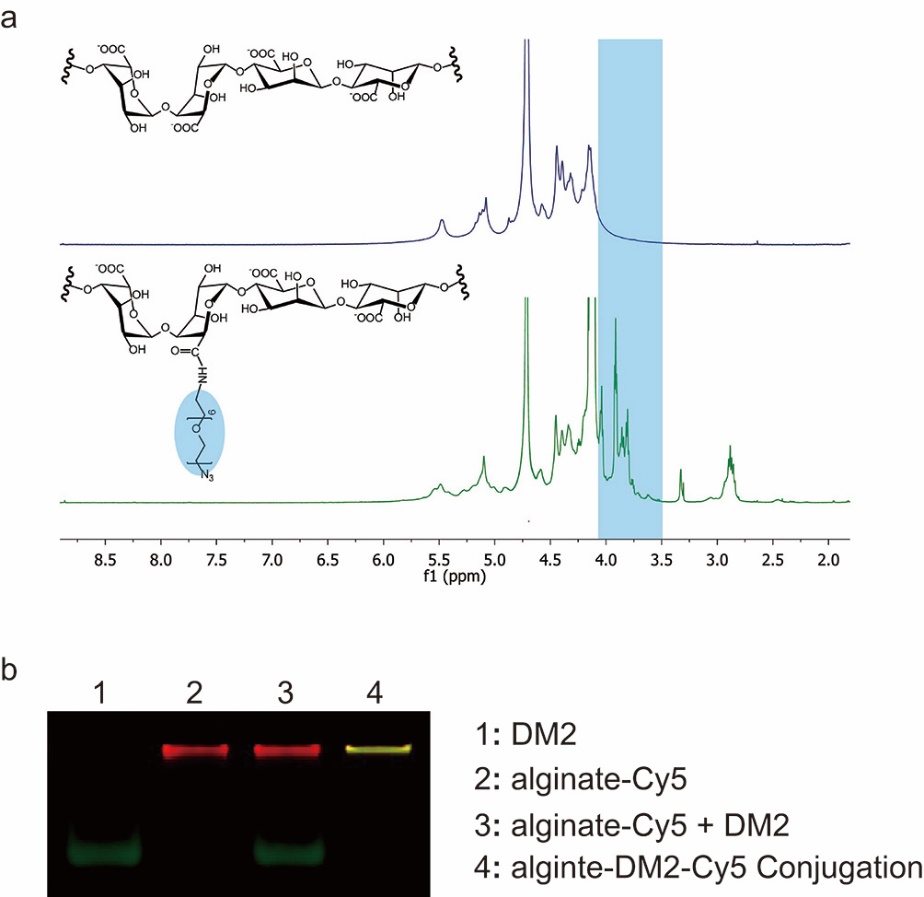


Supplementary Figure 1. Characterization of alginate-DM2 macromer. a) ^1^H NMR spectrum of alginate before and after modification with azide. b) Gel electrophoresis image showing the effective alginate-DM2 conjugation.


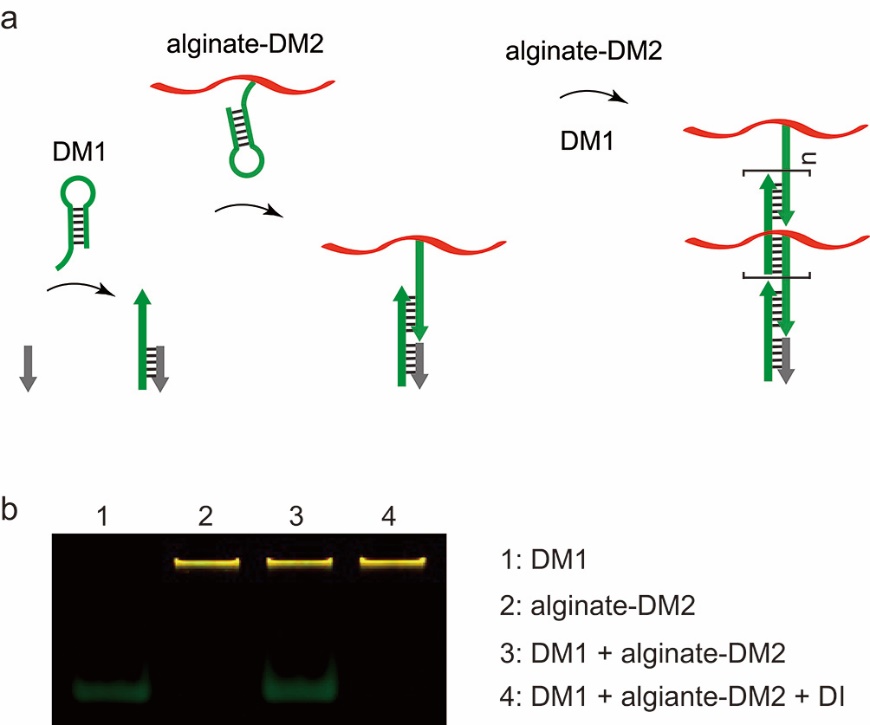


Supplementary Figure 2. Illustration (a) and characterization (b) of DM1 and alginate-DM2 polymerization. The gel image (b) demonstrates the success of the polymerization.


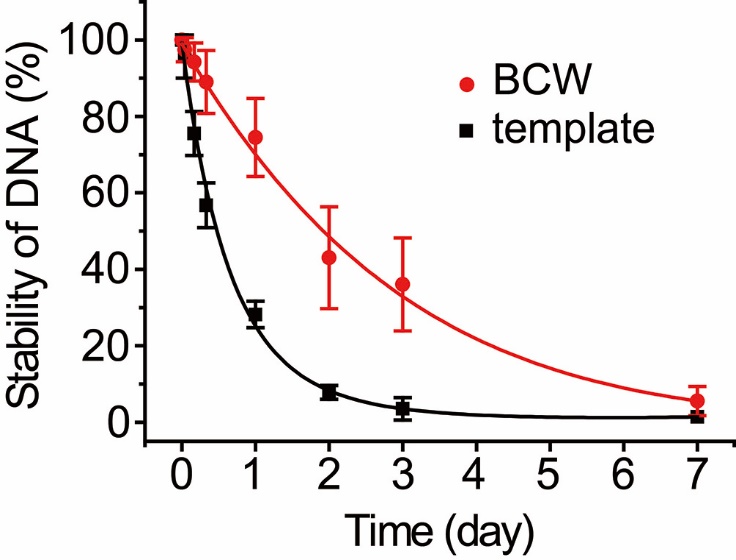


Supplementary Figure 3. Examination of the stability of DNA in the template or BCW on the particle surface. DM1 was labeled with FAM. The flow cytometry analysis was performed to examine the change of the FAM signal (indication of DNA stability) as the function of time. Data are presented as mean ± standard deviation as indicated by error bars (n=3).


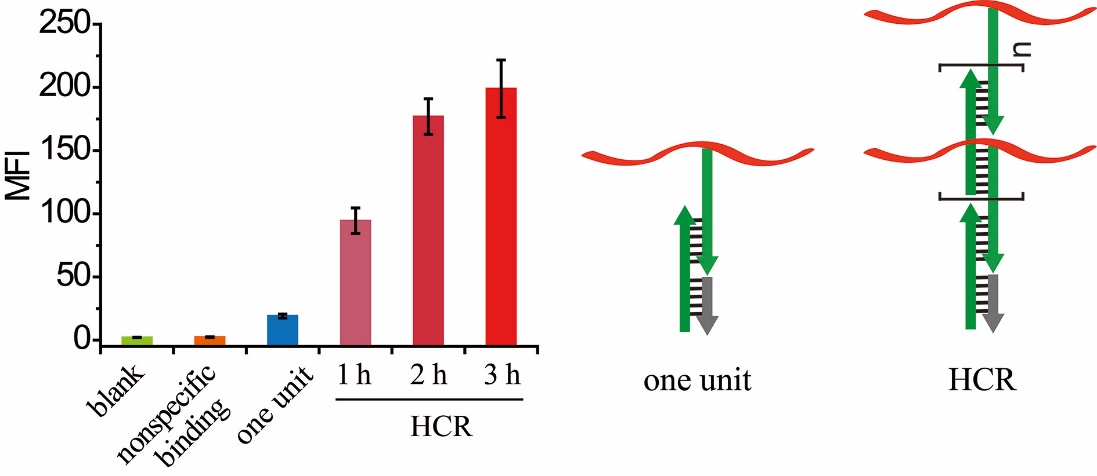


Supplementary Figure 4. Effect of reaction time on the display of supramolecular template on the cell surface. Data are presented as mean ± standard deviation as indicated by error bars (n=3).


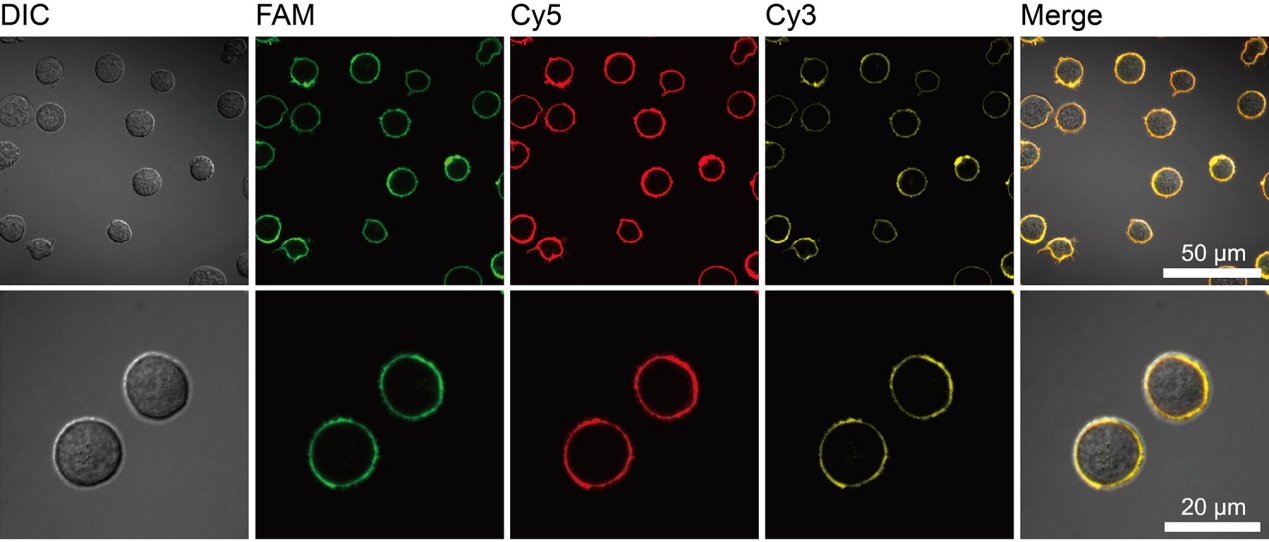


Supplementary Figure 5. Confocal microscopy images of CCRF-CEM cells. DM1, alginate and polylysine were labeled with FAM, Cy5 and Cy3, respectively.


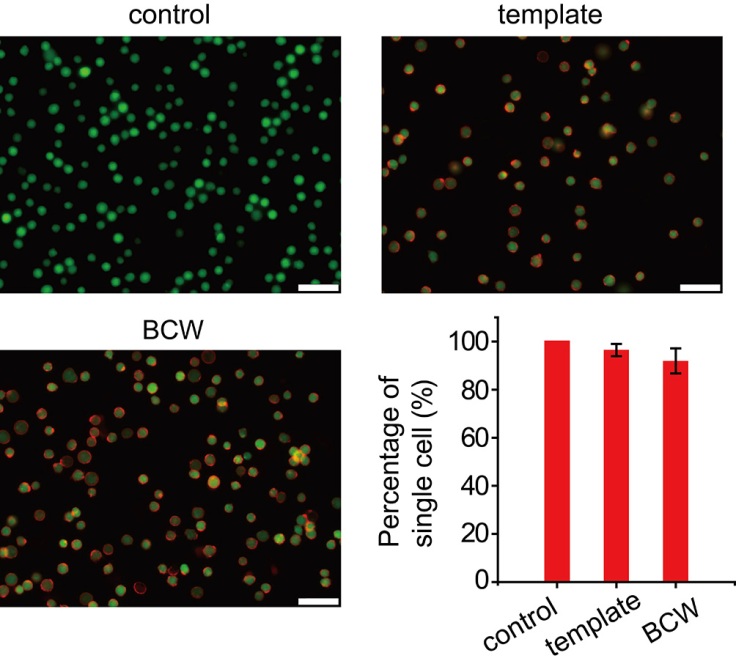


Supplementary Figure 6. Fluorescence imaging for the examination of the yield of single cells. The cells were stained with Calcein-AM (green). Red: Alginate-Cy5. Scale bars: 50 μm. Data are presented as mean ± standard deviation as indicated by error bars (n=8). The images show that the construction of the template and BCW did not cause significant cell aggregation.


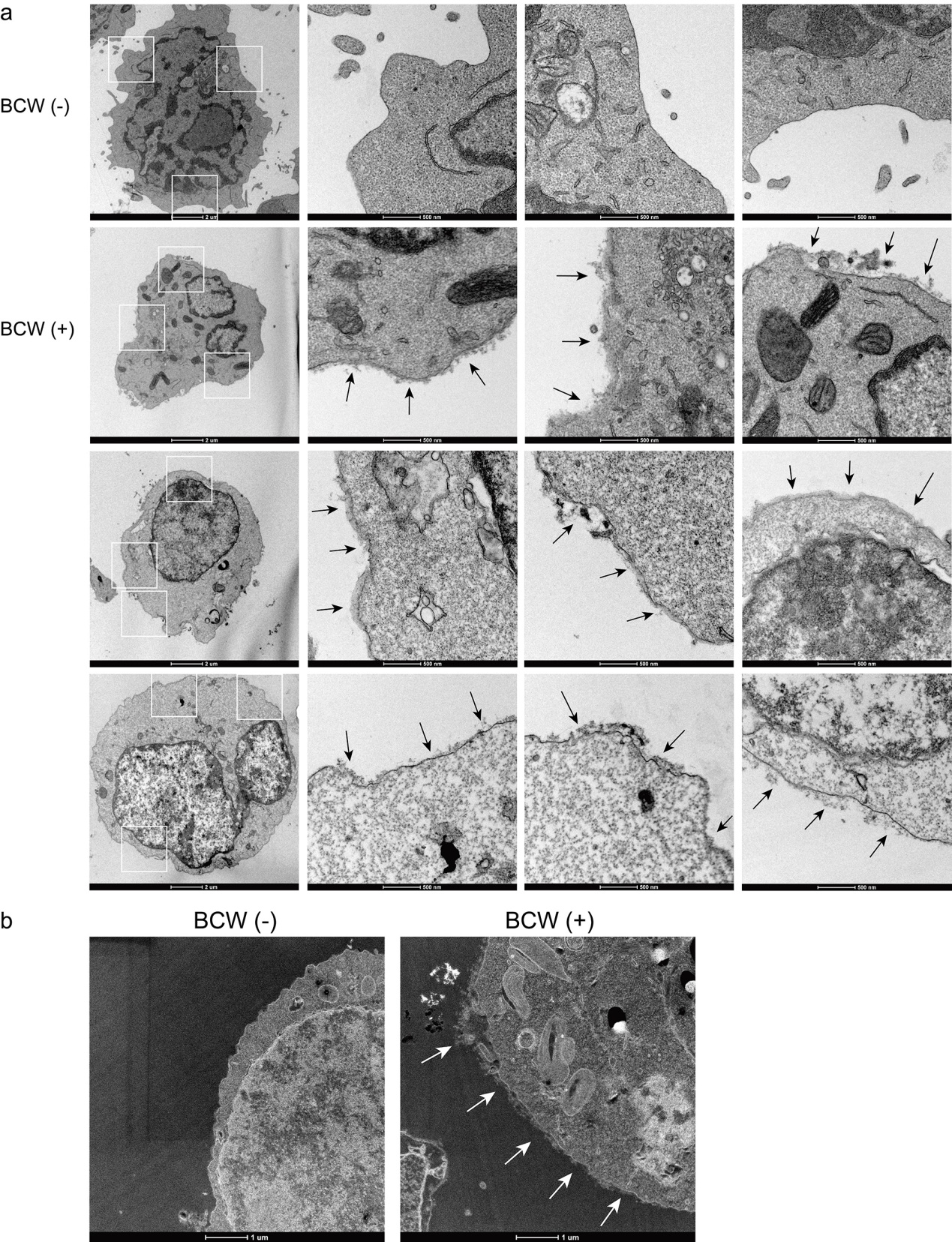


Supplementary Figure 7. Examination of BCW on the cell surface using the TEM (a) and STEM (b) modes. Three BCW-covered cells as labeled with BCW (+) were imaged using the TEM mode (a) and three locations for each cell were chosen for higher magnification. The scale bar is depicted at the bottom of each image. The arrows point to BCW.


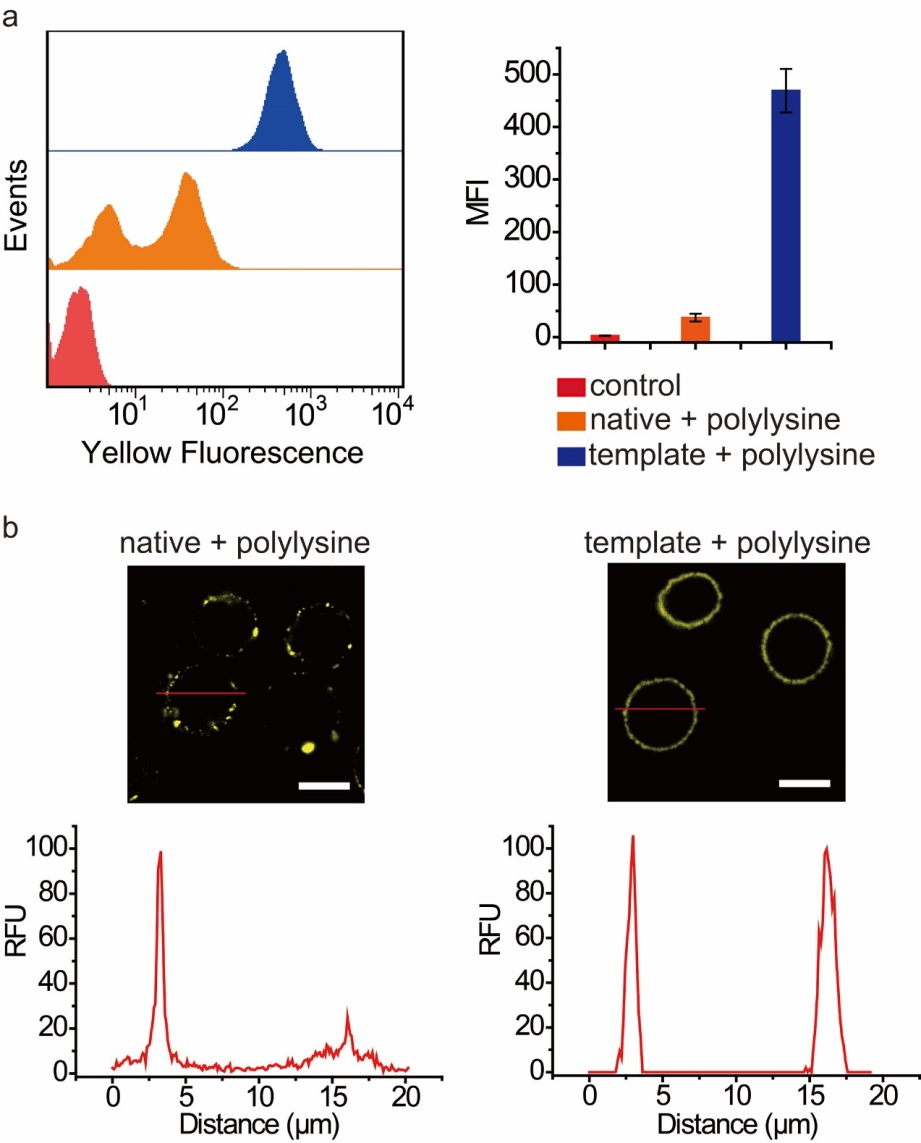


Supplementary Figure 8. Comparison of native and template-covered cells in binding to polylysine. a) Flow cytometry analysis demonstrates that 1) the amount of polylysine attached on the template-covered cells was 13 times more than that on the native cells and 2) the direct binding of polylysine to the native cells was heterogeneous. Data are presented as mean ± standard deviation as indicated by error bars (n=3). b) Confocal microscopy images confirm that the direct binding of polylysine to the native cells was heterogeneous whereas the binding of polylysine to the template-covered cells was relatively much more uniform. Scale bar: 10 μm.


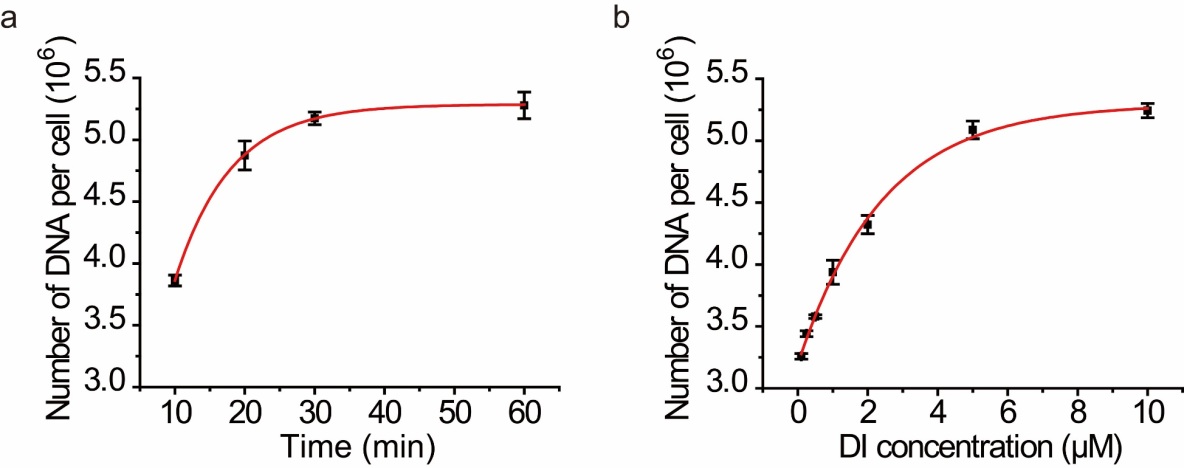


Supplementary Figure 9. Quantitation of DNA initiator (DI) on the cell surface. a) Effect of incubation time on the amount of DI on the cell surface. The concentration of DI was 1 µM. b) Effect of DI concentration on its amount on the cell surface. The incubation time was 30 min. Data are presented as mean ± standard deviation as indicated by error bars (n=3).


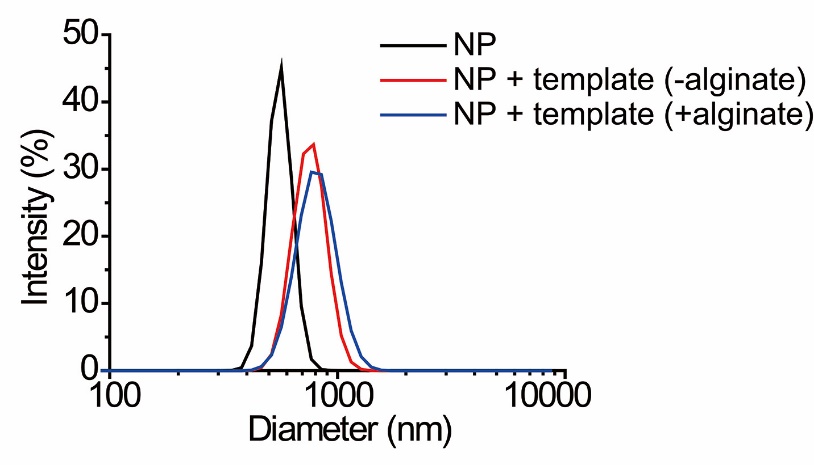


Supplementary Figure 10. Analysis of DNA polymerization (with or without alginate) on the particle surface using dynamic light scattering.


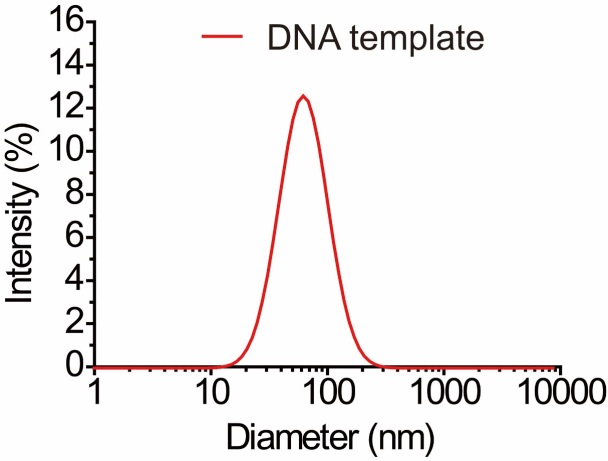


Supplementary Figure 11. Dynamic light scattering analysis of the DNA template.


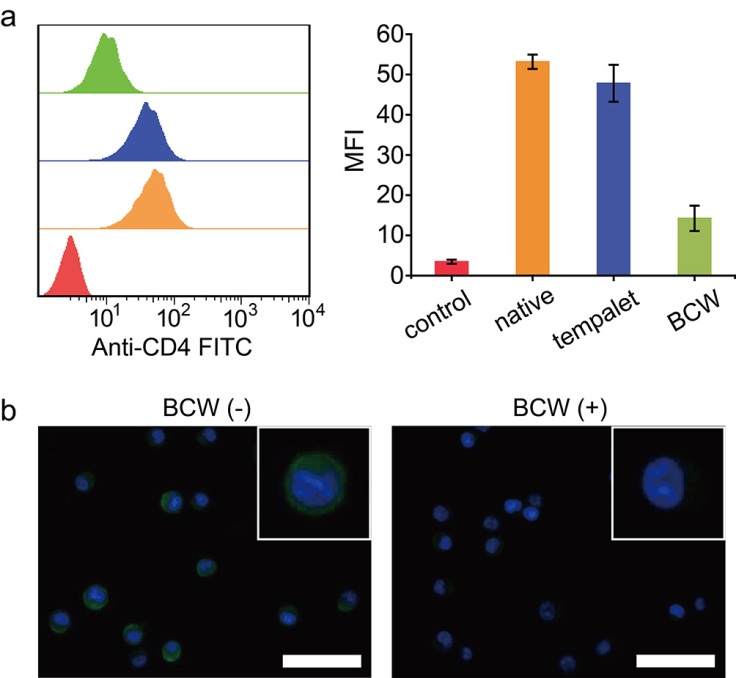


Supplementary Figure 12. Examination of antibody transport across BCW. Flow cytometry analysis (a) and fluorescence imaging (b) of CCRF-CEM cells covered with or without BCW. Green: Anti-CD4-FITC. Blue: DAPI. Scale bars: 50 μm. Data are presented as mean ± standard deviation as indicated by error bars (n=3).


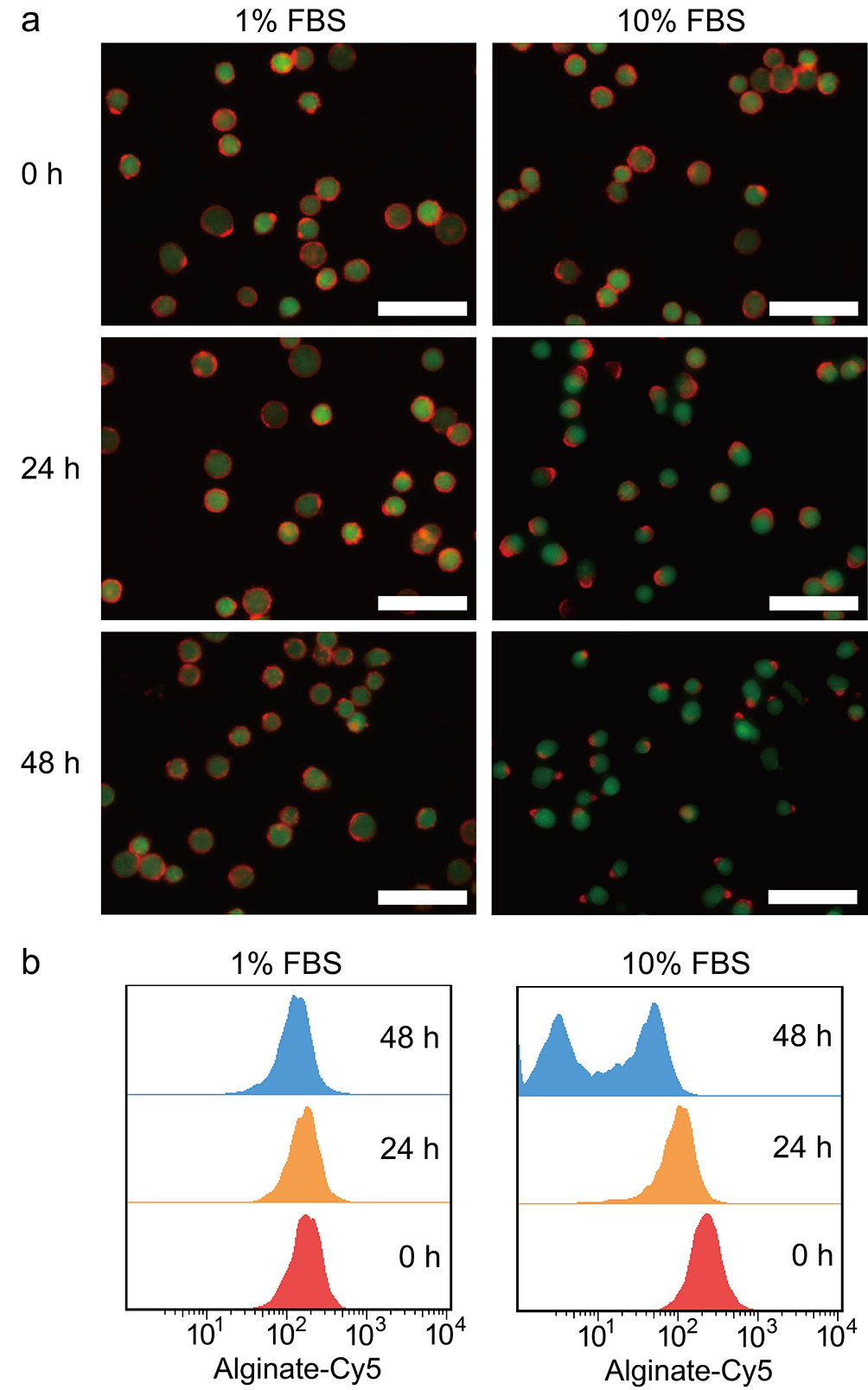


Supplementary Figure 13. Examination of the stability of BCW on CCRF-CEM cells. The cells were cultured in the cell culture media supplemented with 1% or 10% FBS and examined at different time points using fluorescence microscopy (a) and flow cytometry (b). Green: Calcein-AM; Red: Alginate-Cy5. Scale bars: 50 μm.


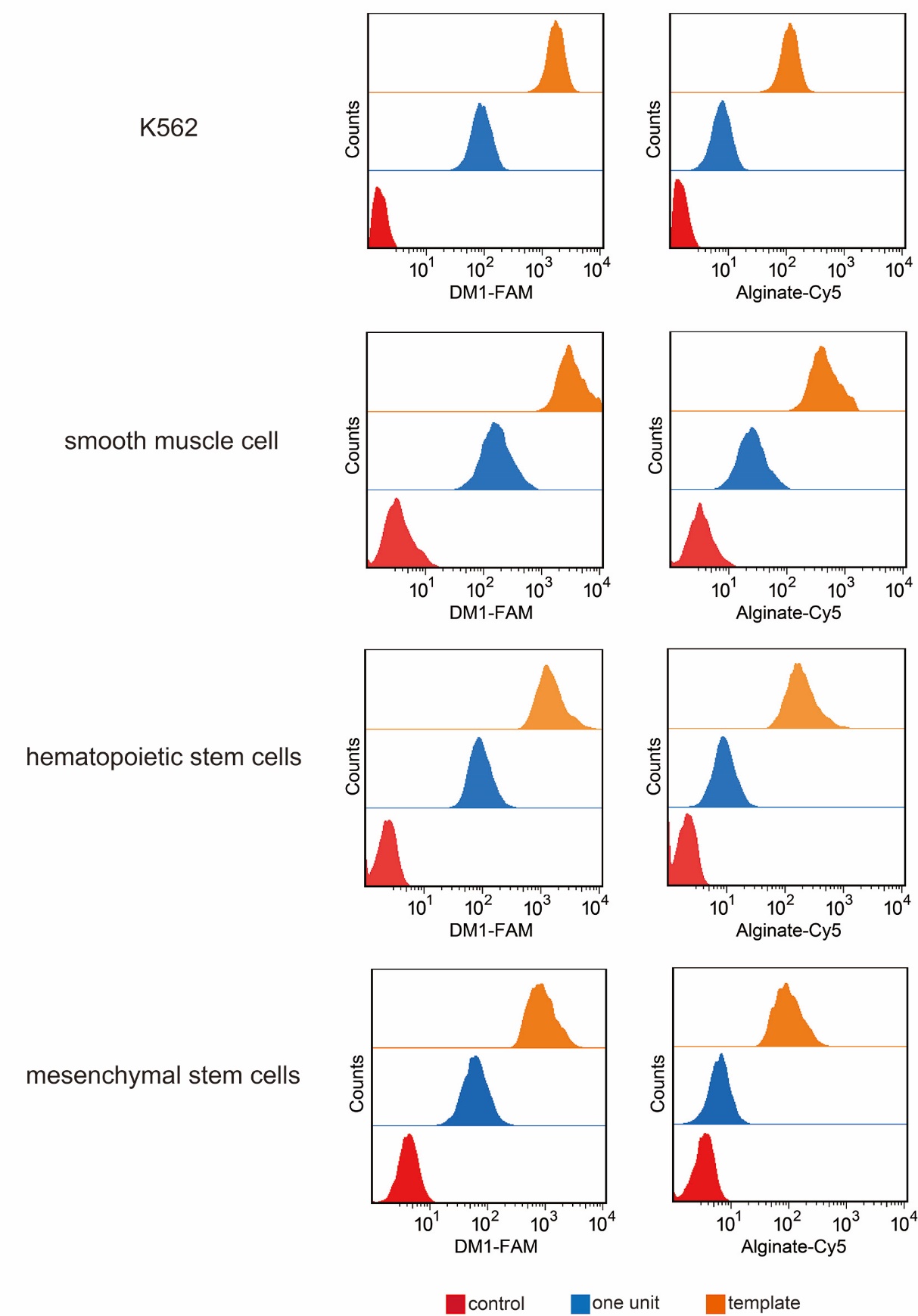


Supplementary Figure 14. Flow cytometry analysis of the template formation on four different types of cells.


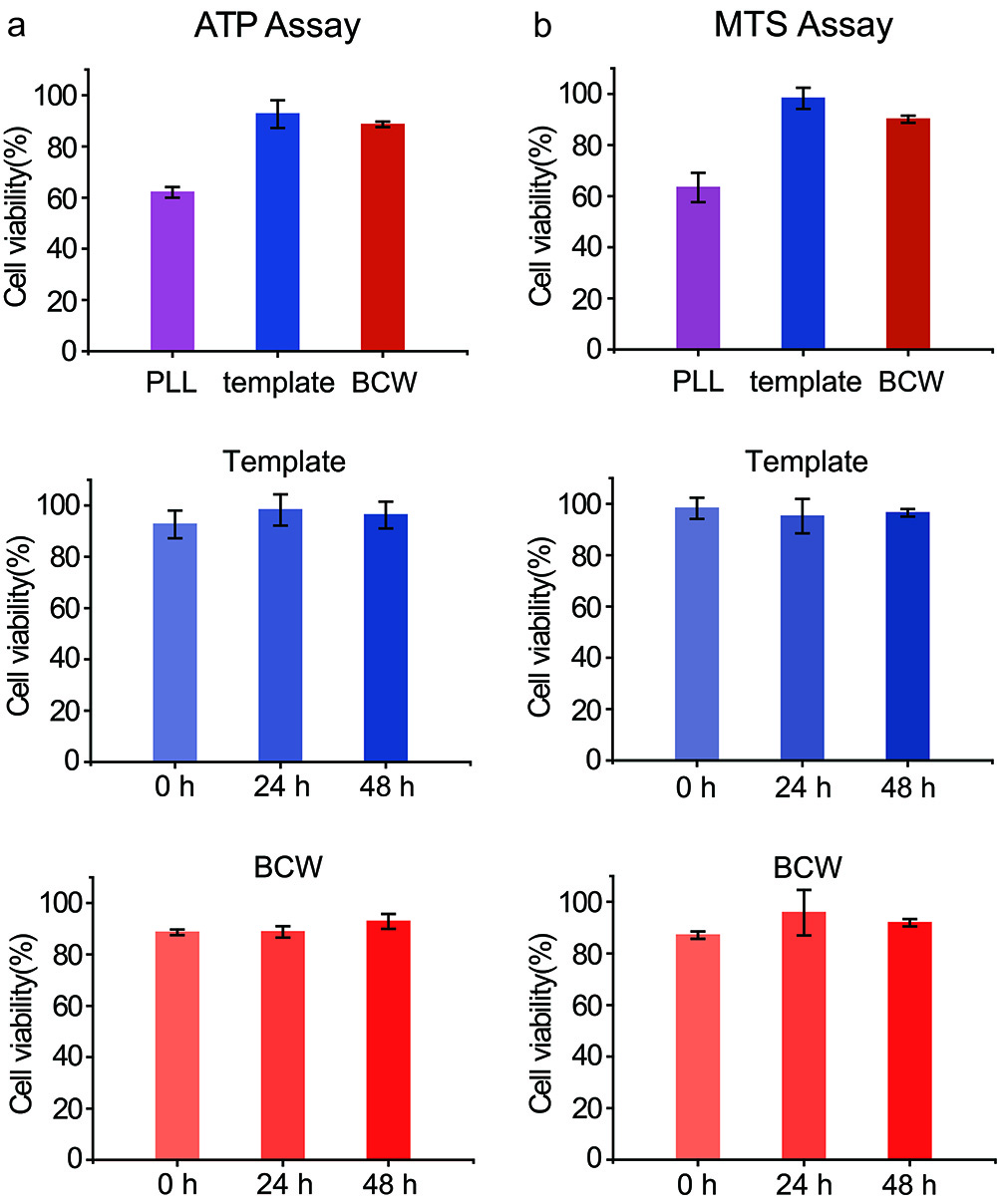


Supplementary Figure 15. Examination of cell viability during and after the construction of the template and BCW. Cell viability was analyzed using ATP (a) and MTS (b) assays. The native cells were used as 100%. The cells were also exposed to polylysine for comparison. Data are presented as mean ± standard deviation as indicated by error bars (n=3).


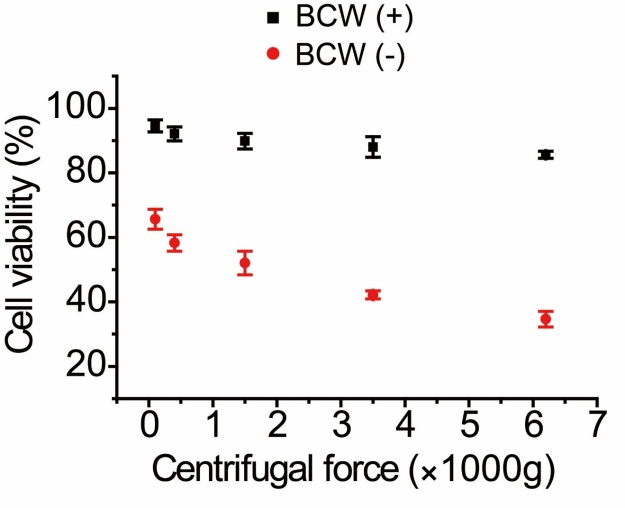


Supplementary Figure 16. Effect of centrifugal force on the viability of cells covered with or without BCW. Data are presented as mean ± standard deviation as indicated by error bars (n=3).


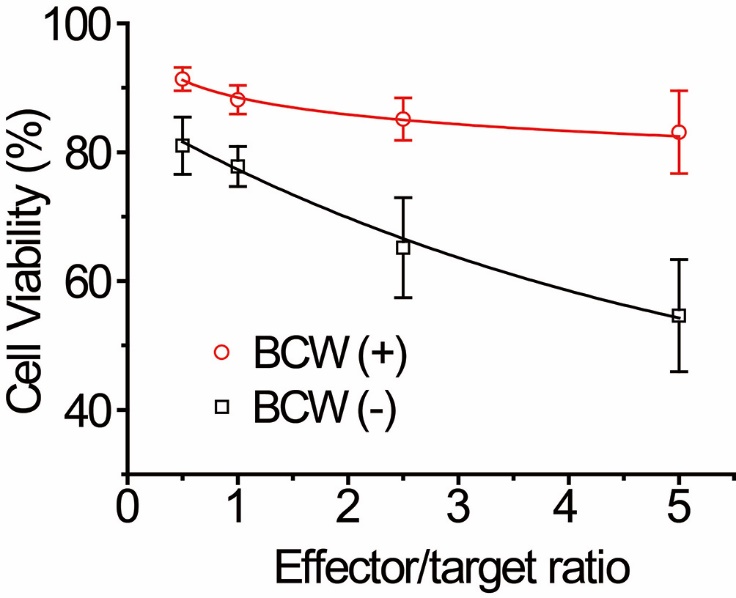


Supplementary Figure 17. Examination of cell protection by BCW from immune attack. Data are presented as mean ± standard deviation as indicated by error bars (n=3). K562 and NK-92MI were used as target and effector cells, respectively.


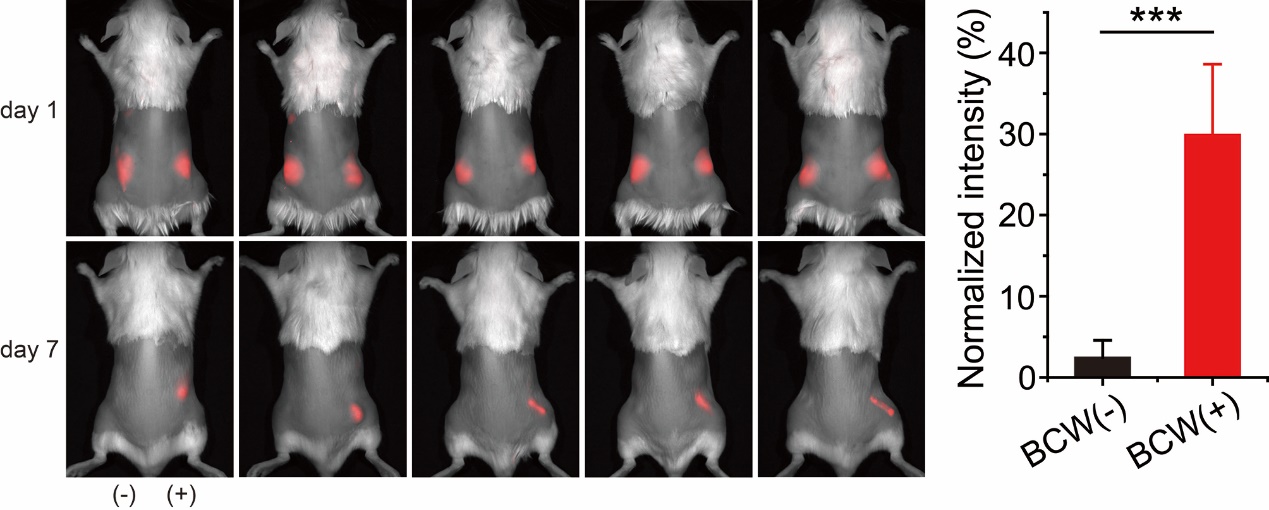


Supplementary Figure 18. In vivo imaging of MSCs covered with (+) or without (-) BCW. The fluorescence intensity at day 7 is normalized to that at day 1 and quantitatively shown in the right figure. Data are presented as mean ± standard deviation as indicated by error bars (n=5). ***, p <0.001. The paired Student’s t test was used to compare the two groups.


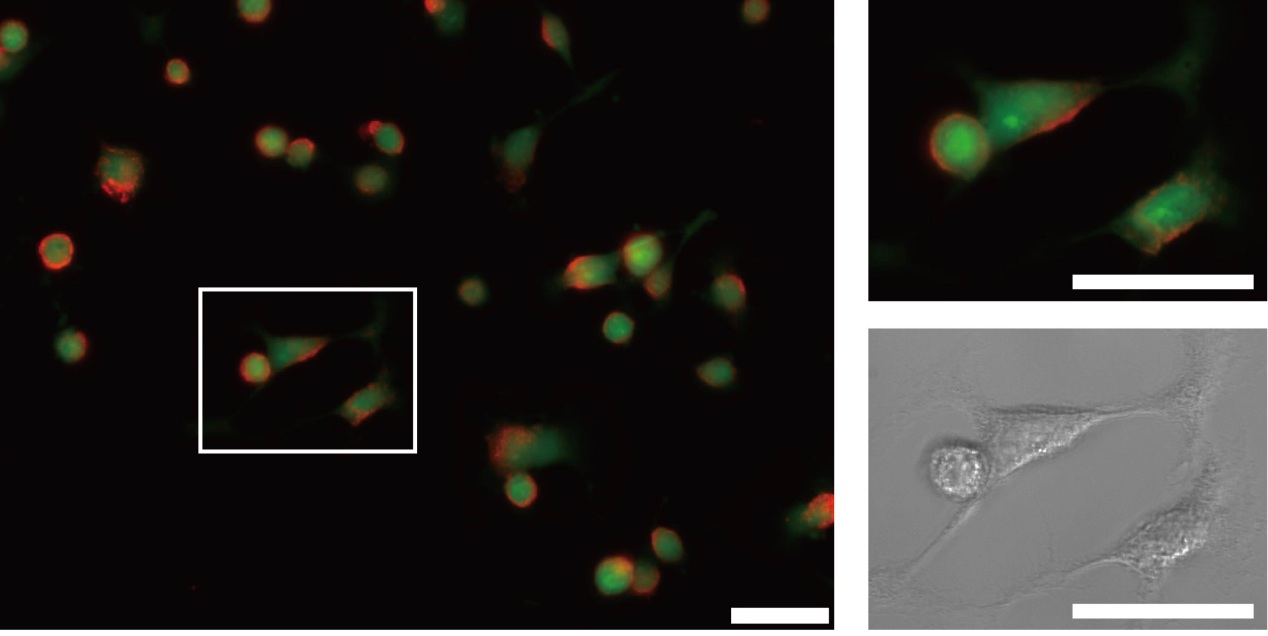


Supplementary Figure 19. Fluorescence imaging of BCW-covered MSCs on the cell culture plate after 2-day culture. Green: Calcein-AM; Red: Alginate-Cy5. Scale bars: 50 μm.


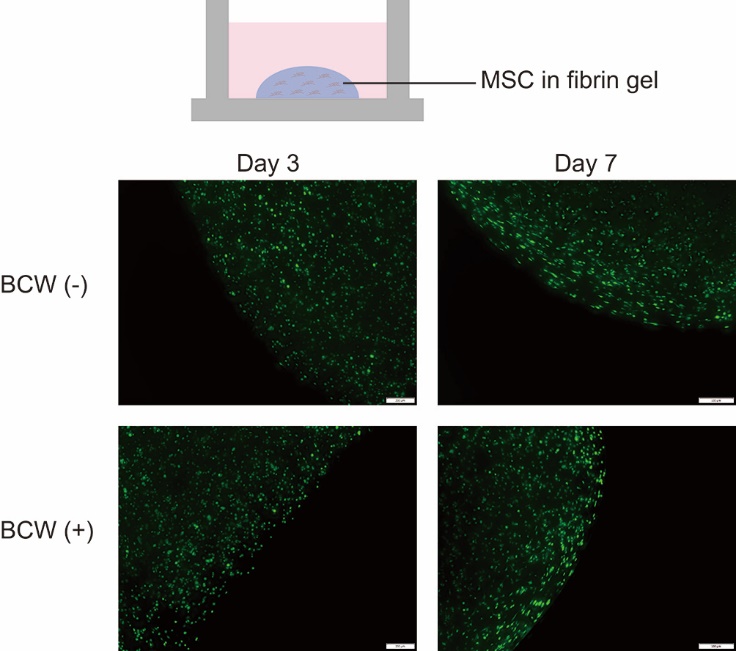


Supplementary Figure 20. Examination of cell retention in fibrin gels. The results show that both BCW-covered and naked MSCs stayed within fibrin gels during the 7-day in vitro culture. Green: Calcein-AM. Scale bars: 200 μm.


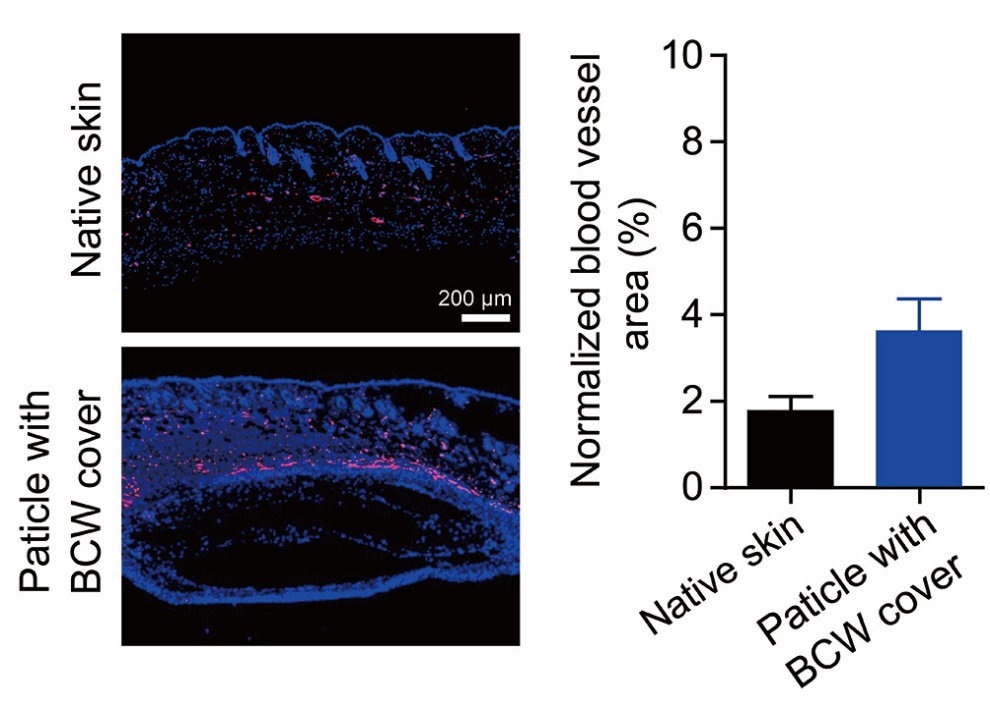


Supplementary Figure 21. Examination of the effect of the alginate-polylysine matrix on angiogenesis. The particles were covered with the alginate-polylysine matrix and implanted subcutaneously. After 10 days, the tissues were collected and stained with the mouse-specific anti-CD31 antibody for the examination of blood vessels. Red: Anti-CD31. Blue: DAPI. Data are presented as mean ± standard deviation as indicated by error bars (n=6).

| **DI concentration** | **Number of DNA /cell** | **The average distance between two adjacent DNA** |
| --- | --- | --- |
| 0.1 μM | 3.3 x 10^6^ | 12.7 nm |
| 0.25 μM | 3.5 x 10^6^ | 10.8 nm |
| 0.5 μM | 3.6 x 10^6^ | 9.7 nm |
| 1 μM | 3.9 x 10^6^ | 8.2 nm |
| 2 μM | 4.3 x 10^6^ | 7.7 nm |
| 5 μM | 5.1 x 10^6^ | 7.3 nm |
| 10 μM | 5.2 x 10^6^ | 6.5 nm |

Supplementary Table 1. Calculation of the average distance between two adjacent DNA templates. The distance was calculated using the *imdistline* matlab function. 10 pairs of adjacent dots were randomly chosen to calculate the average distance. Specifically, the cell was assumed as a sphere with a radius of 6μm to calculate the total surface area of a cell. As one DNA initiator was assumed to induce the formation of one DNA template, we got N templates randomly distributed on the cell surface. One dot represented one DNA initiator or template. Thus, N dots represented how many DNA initiators or templates were distributed onto the surface area of a cell in the calculation.

| **Osmotic**  **imbalance**  **(∆P/P^0^)** | **Cell viability (%)** | |
| --- | --- | --- |
|  | BCW(+) | BCW(-) |
| 1/9 | 98.6±0.05 | 95.4±0.6 |
| 2/9 | 97.5±4.2 | 92.0±2.8 |
| **3/9** | **79.0±1.7** | **60.3±2.6** |
| **4/9** | **69.0±1.9** | **38.8±0.2** |
| **5/9** | **50.0±3.1** | **21.8±5.2** |
| **6/9** | **37.6±0.5** | **17.1±1.7** |
| 7/9 | 11.3±4.9 | 7.0±2.0 |
| 8/9 | 5.9±0.9 | 5.8±1.8 |

Supplementary Table 2. Effect of osmotic imbalance on the viability of cells covered with or without BCW. For the clear legibility of shielding enhancement, the middle groups were highlighted with red. Data were presented as mean±s.d, n=3.

| **DNA name** | **Sequence (5-->3)** |
| --- | --- |
| **Cholesterol-TEG-DI** | CCTCATCCCACTCCTACCTAAACCAAAAAAAAAA/3CholTEG/ |
| **DI-Biotin** | CCTCATCCCACTCCTACCTAAACCAAAAAAAAAA/3Bio/ |
| **DM1** | GGTTTAGGTAGGAGTGGGATGAGGCCAAATCCTCATCCCACTCCTACC |
| **DM1-FAM** | GGTTTAGGTAGGAGTGGGATGAGGCCAAATCCTCATCCCACTCCTACC**/36-FAM/** |
| **DM2-NH_2_** | **/5AmMC6/**AAAAACCTCATCCCACTCCTACCTAAACCGGTAGGAGTGGGATGAGGATTTGG |

Supplementary Table 3. DNA sequences.
